# MultiCausGRN: directed prior-guided graph attention model for multi-omics gene regulatory network inference

**DOI:** 10.3389/fbinf.2026.1883130

**Published:** 2026-08-04

**Authors:** Noor Jamal Alkhateeb, Mamoun Awad

**Affiliations:** College of IT, UAE University, Al Ain, United Arab Emirates

**Keywords:** gene regulatory networks, graph attention networks, multi-omics data, bioinformatics, single cell RNA sequence, chromatin accessibility

## Abstract

**Introduction:**

Existing methods for gene regulatory network (GRN) inference rely primarily on gene expression data alone or on lower-resolution bulk sequencing data. Despite recent advances in integrating chromatin accessibility and RNA sequencing, inferring GRNs from paired single-cell multi-omics data remains challenging due to noise, sparsity, and complex nonlinear regulatory relationships.

**Methods:**

We present MultiCausGRN, a graph attention network (GAT)-based framework for GRN inference from paired scRNA-seq and scATAC-seq data. The model incorporates directed prior-guided graph attention learning to capture biologically grounded regulatory directionality by integrating curated directed regulatory edges into graph representation learning. MultiCausGRN performs supervised transcription factor–target link prediction using integrated multi-omics features within a two-layer graph attention architecture.

**Results:**

On the human PBMC multi-omics dataset, prior knowledge integration improved predictive stability and achieved a mean test AUPRC of 0.743 ± 0.049 and a mean AUROC of 0.682 ± 0.026 across five independent random seeds.

**Discussion:**

These results demonstrate that directed prior-guided graph learning can improve the robustness and biological interpretability of GRN inference in data-limited settings. MultiCausGRN is publicly available at: https://github.com/nrr-90/MultiCausGRN.

## 1 Introduction

Gene Regulatory Networks (GRNs) are collections of molecular regulators that interact with each other and determine gene activation and silencing in specific cellular contexts (Hecker et al., 2009). A comprehensive understanding of gene regulation is fundamental to explaining how cells perform diverse functions, how cells alter gene expression in response to different environments, and how non-coding genetic variants cause disease. GRNs are composed of transcription factors (TFs) that bind DNA regulatory elements to activate or repress the expression of target genes (TG) (Alkhateeb and Awad, 2024; Alkhateeb and Awad, 2025). Inference of GRNs is a central problem (Badia Mompel et al., 2023), and there have been many attempts to approach it (Zhong et al., 2020; Dong et al., 2024). Recent advances in biochemistry and sequencing technologies have enabled the monitoring of biological systems at an unprecedented resolution, leading to the development of GRN inference methods for both bulk sequencing (Kovarik et al., 2012; Emma Paul et al., 2024; Ferebee and Buckler, 2023), and single-cell RNA sequencing (scRNA-seq) data (Kolodziejczyk et al., 2015). Bulk sequencing-based GRN inference has been widely used to analyze gene interactions across different conditions, offering valuable insights into regulatory mechanisms at the tissue level. However, bulk sequencing averages gene expression across cell populations, making it insufficient for capturing cell-type-specific regulatory interactions and dynamic changes in heterogeneous systems. In contrast, scRNA-seq-based GRN inference enables the dissection of complex tissues into individual cell types, allowing for the study of gene regulation at single-cell resolution. This approach provides deeper insights into cell differentiation, lineage specification, and dynamic regulatory processes. Inferring regulatory relationships between genes using transcriptomics data alone is highly challenging (Hecker et al., 2009). One major limitation is that transcriptomics data cannot distinguish between direct and indirect regulatory interactions, which inevitably leads to the introduction of false positives into the inferred networks (Hetzel et al., 2021). To address this issue, DeepTFni (Li et al., 2022) infers gene regulatory relationships using chromatin accessibility data. Chromatin accessibility analysis enables the identification of direct regulatory interactions, as active regulatory elements for TF binding and gene regulations are generally accessible (Grandi et al., 2022). Experimental results from DeepTFni demonstrate that chromatin accessibility data could also provide regulatory information between genes. With the development of single-cell technologies, single-cell multi-omics (scMulti-omics) techniques allow us to observe multimodal data within the same cell, which provides new opportunities to reproduce cell types and gene regulation more comprehensively (Badia Mompel et al., 2023). In the field of cell type inference, several studies have found that analyzing transcriptomics data in conjunction with chromatin accessibility data allows for the discovery of additional cell subtypes (Liu et al., 2025). Deep learning approaches in GRN inference typically reformulate network reconstruction as a supervised (Emma Paul et al., 2024; Mao et al., 2023) or semi-supervised learning problem (Wang et al., 2021). Many computational methods have been developed to infer GRNs from gene expression data, predominantly using unsupervised techniques. Unsupervised methods rely on expression data only but tend to achieve lower prediction accuracies than supervised methods (Prill et al., 2010). However, several unsupervised learning methods use multi-omics data to infer GRNs. scMTNI (Zhang et al., 2023) and SCENIC+ (Bravo González-Blas et al., 2023) first identify TF binding sites by performing motif analysis in accessible chromatin (ATAC peak) regions to construct initial TF–gene or TF–cis-regulatory element (CRE) relationships. MTLRank (Song et al., 2023) computes TF activity scores from ChIP-seq and scATAC-seq data to estimate the regulatory effects between each TF and gene in the cell, and combines TF activity with TF expression to predict RNA velocity using a multi-layer neural network. These studies have all found that using gene expression and chromatin accessibility data for GRN inference can better identify direct and indirect regulatory relationships, reduce false positives and false negatives, and improve accuracy. However, they are affected by the curse of dimensionality when dealing with a large number of genes. By contrast, supervised methods require information about known interactions for training, and this information is typically sparse. CNNC (Yuan and Bar-Joseph, 2019) a supervised learning framework, learns from prior knowledge and infers GRNs using deep convolutional neural networks. Semi-supervised methods reflect a compromise and can be trained with much less interaction data, but usually are not as accurate predictors as supervised methods. Authors in (Mordelet and Vert, 2008) evaluated supervised inference of regulatory networks (SIRENE) in comparison to context likelihood of relatedness (CLR), algorithm for the reconstruction of accurate cellular networks (ARACNE), relevance networks (RN), and a Bayesian network (BN) on an *Escherichia coli* benchmark data set by Faith et al. (2007). They found that the supervised method considerably outperformed the unsupervised techniques. Cerulo et al. (2010) compared supervised and semi-supervised support vector machines (SVMs) with two unsupervised methods and found the former superior. In recent years, an increasing number of supervised methods for GRN inference have been proposed (Chan et al., 2017; Pratapa et al., 2020). However, they rely solely on gene expression data, without incorporating additional biological information. This limitation can lead to false positives in GRN inference due to the single modality approach.

Many methods integrate unpaired scRNA-seq and scATAC-seq data to infer trans-regulation. Recent multi-omics technologies enable simultaneous measurement of gene expression and chromatin accessibility, providing complementary regulatory signals. Several GRN inference approaches integrate these modalities to reduce indirect correlations and improve the detection of direct regulatory interactions. Those methods, including IReNA (Jiang et al., 2022), SOMatic (Jansen et al., 2019), UnpairReg (Yuan and Duren, 2022), CoupledNMF (Duren et al., 2018), and others (Wang et al., 2020), link TFs to regulatory elements (RE) by motif matching and link REs to TGs using the covariation of RE–TG or physical base pair distance.

Despite recent progress, existing multi-omics GRN inference methods present several limitations. Some approaches rely on large external reference atlases or extensive motif-based prior construction, which may restrict applicability in data-limited scenarios. Others focus primarily on multi-modal feature fusion without explicitly modeling prior knowledge regulatory structure. Furthermore, robustness and stability across different random initializations remain underexplored. Although single-cell data offers a large number of cells, most of them are not independent. Finally, incorporating prior knowledge, such as motif matching, into non-linear models is challenging. To overcome these challenges, we propose MultiCausGRN, a prior-guided graph attention model for multi-omics GRN inference. The term directed prior refers to biologically curated directed regulatory interactions obtained from prior knowledge resources. These priors are used to guide graph construction and message passing and should not be interpreted as formal causal inference or causal discovery. The proposed model integrates transcriptional and chromatin accessibility features within a supervised graph attention network (GAT) architecture, while incorporating biologically grounded transcription factor–target prior edges as directional constraints during message passing. Unlike existing multi-omics GRN methods that rely primarily on feature fusion, motif filtering, or external atlas pretraining, MultiCausGRN integrates filtered TF–target priors directly into graph attention message passing while preventing validation/test leakage. This enables biologically constrained nonlinear representation learning for GRN inference from paired single-cell multi-omics data. The main contributions of this work are threefold: (1) integrating prior knowledge into graph attention learning for GRN inference; (2) demonstrating improved robustness and stability on PBMC multi-omics data; and (3) providing a structured comparison with recent multi-omics GRN inference methods. The remainder of the paper is organized as follows: Section 2 presents the materials and methods. Results are discussed in Section 3, and Section 4 presents the conclusion.

## 2 Materials and methods

Link prediction is a fundamental problem in biological network science that estimates the likelihood of interactions between pairs of biological entities (Zhang and Chen, 2018). The process of supervised link prediction is to learn a mapping function that encodes nodes into dense vectors while preserving intrinsic properties (Chan et al., 2017). The vectorized representations learned for nodes can be used to predict potential links via some score functions (Zhang and Chen, 2018). We formulate GRN inference as a supervised link prediction task between transcription factors and target genes. MultiCausGRN integrates multi-omics node features within a graph attention network while incorporating biologically grounded prior edges to guide message passing.

### 2.1 The MultiCausGRN model

MultiCausGRN regards the GRN inference task as a link prediction problem in a gene network that discovers the latent interactions between node pairs, where the nodes are the genes, edges are TF-targets, features can be scRNA or scATAC sequence data, and the ground-truth database is OmniPath V1 (November 2016) (Türei et al., 2016). MultiCausGRN projects gene expression profiles onto a low-dimensional space using a knowledge-based interaction matrix, assuming that the generative process of the true data is that of gene regulation (Hetzel et al., 2021). The goal of MultiCausGRN is to optimize the embedding space to learn high-quality gene representations for predicting potential regulatory relationships between TFs and genes. Given the multi-omics gene expression matrix $X=\left[X_{\mathrm{RNA}}\|X_{\mathrm{ATAC}}\right]\in R^{N\times 2C}$ , where $X_{\mathrm{RNA}}\in R^{N\times C}$ , $X_{\mathrm{ATAC}}\in R^{N\times C}$ , the operator ‖ denotes vector concatenation, C is the number of cells, and N is the number of genes, and with the associated adjacency matrix $A\in R^{N\times N}$ of a prior network, MultiCausGRN expects to obtain the initial node representations $H^{\left(0\right)}$ by applying a linear transformation followed by a nonlinear activation function as in Equation 1.

$$H^{\left(0\right)}=\sigma \left(XW_{f}+b_{f}\right)$$ (1)

where σ is the activation function, $W_{f}$ is a learnable weight matrix, and $b_{f}$ is a bias vector. MultiCausGRN comprises two GAT layers followed by two MLP channels. By jointly using different types of neural networks, MultiCausGRN can learn robust, generalizable gene representations to measure similarity or infer the directed regulatory likelihood between two nodes. In the first GAT layer, each node shares a parametrized weight matrix $W^{F^{\prime }\times F}$ , where F is the number of features per gene and F’ is the number of new features produced by the layer. Then, the self-attentional mechanism is applied to the gene representations to obtain the attention coefficients, as given in Equation 2. The function LeakyReLU introduces nonlinearity into the attention computation; hence, Equation 2 represents the unnormalized attention score measuring the importance of node j to node i . To make coefficients easily comparable across different nodes, we normalize them across all choices of j using the softmax function as in Equation 3, (Veličković et al., 2018).

$$e_{ij}^{\left(l\right)}=\text{LeakyReLU}\left(a^{\left(l\right)T}\left[W^{\left(l\right)}h_{i}^{\left(l\right)}\|W^{\left(l\right)}h_{j}^{\left(l\right)}\right]\right)$$ (2)

$$\alpha_{ij}^{\left(l\right)}=\frac{exp\left(e_{ij}^{\left(l\right)}\right)}{\underset{k\in N\left(i\right)}{\sum }exp\left(e_{ik}^{\left(l\right)}\right)}$$ (3)

Where $h_{i}^{\left(l\right)}$ denotes the feature representation of node i at layer l , $W^{\left(l\right)}$ is a learnable weight matrix, $a^{\left(l\right)}$ is the attention weight vector, the operator ‖ denotes vector concatenation, and N(i) denotes the set of neighboring nodes of node i in the graph. Note that the attention coefficient $\alpha_{ij}$ can be viewed as the regulatory strength between gene i and gene j . These coefficients represent the normalized importance of each neighbor during message aggregation. The updated representation of node i is computed using Equation 4 as a weighted aggregation of its neighbors’ transformed features, where σ is a nonlinear activation function, and the weights are given by the attention coefficients. This allows the model to selectively focus on more relevant regulatory interactions.

$$h_{i}^{\left(l+1\right)}=\sigma \left(\underset{j\in N\left(i\right)}{\sum }\alpha_{ij}^{\left(l\right)}W^{\left(l\right)}h_{j}^{\left(l\right)}\right)$$ (4)

Multi-head attention mechanisms are applied to benefit robust node representations, see Equation 5. The first graph attention layer employs multi-head attention with feature concatenation, while the second layer aggregates heads by averaging to produce final node embeddings. K independent attentions are concatenated to get the output gene representations of this layer:

$$h_{i}^{\left(l+1\right)}={\|}_{k=1}^{K}\sigma \left(\underset{j\in N\left(i\right)}{\sum }\alpha_{ij}^{k}W^{k}h_{j}^{\left(l\right)}\right)$$ (5)

Where, k is concatenation operation; $\alpha_{ij}^{k}$ is the $k_{th}$ normalized attention coefficient; $W^{k}$ is the transposed weight matrix for linear transformation; σ is RELU (exponential linear unit) function. In the second GAT layer, the average of the multi-head attentions is computed as in Equation 6:

$$z_{i}=\sigma \left(\frac{1}{K}\underset{k=1}{\overset{K}{\sum }}\underset{j\in N_{i}}{\sum }\alpha_{ij}^{k}W^{k}h_{j}\right)$$ (6)

where $z_{i}$ represents the final embedding of node i . In the final layer, outputs from multiple attention heads are averaged instead of concatenated to produce a stable representation for downstream prediction. Out of the GAT layers, pairwise genes i and j are input into two channels with the same structure. Each channel is composed of MLPs to further encode representations into low-dimensional vectors, see Equation 7. Finally, the dot product is used as the score function to evaluate the similarity of this pair of genes based on learned representations as seen in Equations 8, 9.

$$u_{t}=MLP_{TF}\left(z_{t}\right),\;v_{g}=MLP_{TG}\left(z_{g}\right)$$ (7)

$$s_{tg}=u_{t}^{T}v_{g}$$ (8)

$${\overset{\wedge }{y}}_{tg}=\sigma \left(s_{tg}\right)$$ (9)

Where $z_{t}$ and $z_{g}$ denote the embeddings of transcription factor t and target gene g , respectively. Two separate multilayer perceptrons $\left(MLP_{TF}\right.$ and $\left.MLP_{TG}\right)$ are used to transform TF and target representations. The interaction score $s_{tg}$ is computed as the dot product between transformed embeddings, and the predicted probability ${\overset{\wedge }{y}}_{tg}$ is obtained by applying a sigmoid activation function σ . The objective of MultiCausGRN is to optimize the pointwise binary cross-entropy (BCE) loss, Equation 10.

$$BCE=-\frac{1}{N}\overset{K}{\underset{i=1}{\sum }}\left[y_{i}log\left(\overset{\wedge }{y_{i}}\right)+\left(1-y_{i}\right)log\left(1-\overset{\wedge }{y_{i}}\right)\right.$$ (10)

Where N denotes the training dataset of TF–target pairs, y is the ground-truth label indicating whether a regulatory interaction exists, and ${\overset{\wedge }{y_{i}}}_{tg}$ is the predicted probability for TF-traget pair i . The model is trained using binary cross-entropy loss to distinguish positive regulatory interactions from negative samples. During training, prior edges are incorporated into the adjacency matrix, biasing attention weights toward biologically plausible regulatory directions. The probability of a regulatory interaction between gene i and gene j is computed using the dot product of their final embeddings, followed by an MLP decoder.

**FIGURE 1:**
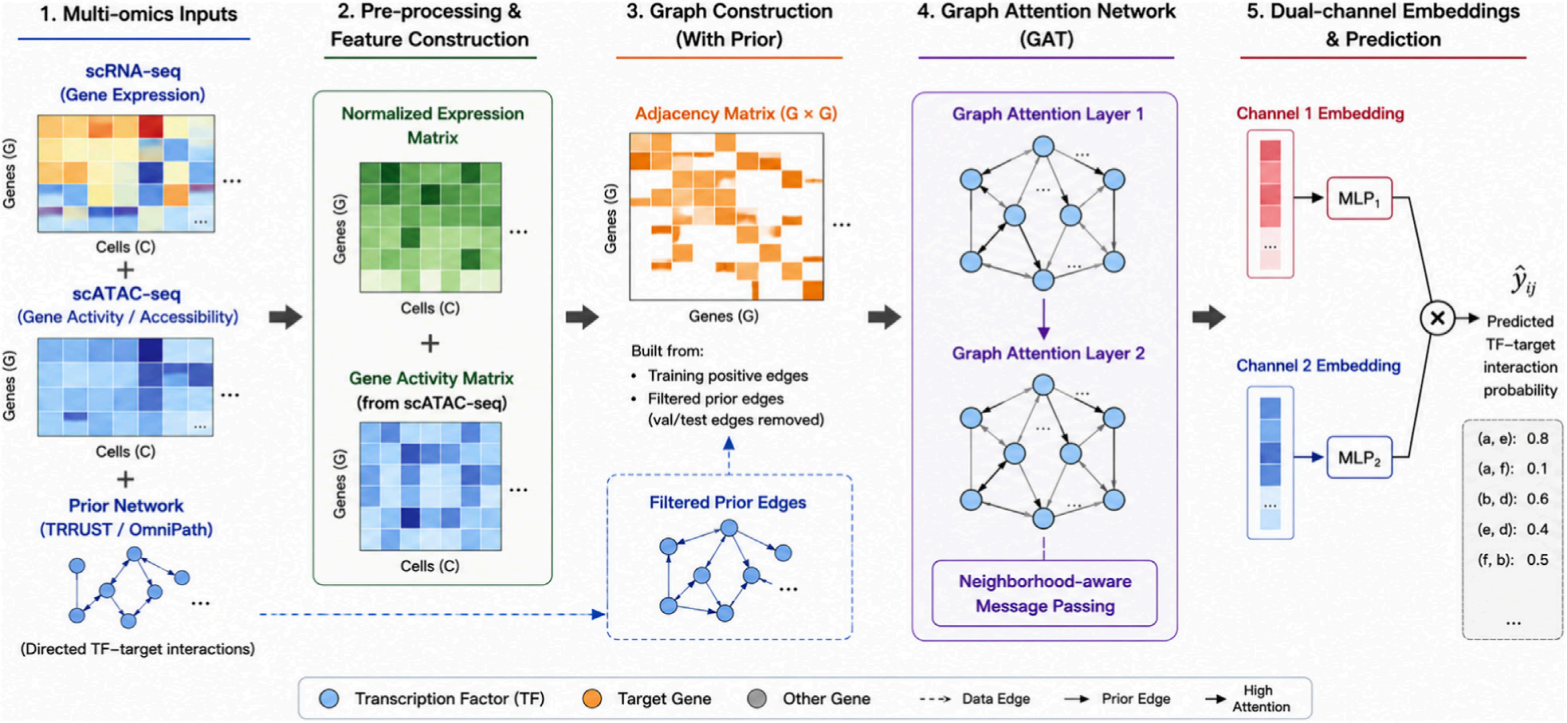
Overview of the MultiCausGRN model. MultiCausGRN is designed with two GAT layers followed by MLPs to learn gene low-dimensional representations. It needs to simultaneously input single-cell gene expression data, scATAC-seq, and prior adjacency matrix. Attention mechanisms are applied to assign implicit weights to neighbour nodes. Meanwhile, we stack two GAT layers to capture higher-order neighbour information. After GAT layers, we separately input pairwise genes, i and j, into two different channels. Both channels are composed of multilayer neural networks that generate dense gene representations ultimately. We apply the dot product as the score function to evaluate the similarity of gene i and gene j.

We integrate biologically grounded prior edges directly into graph attention learning as in Equations 11–13.

$$\overset{*}{\underset{prior}{E}}=E_{\mathrm{prior}}\backslash \left(E_{\mathrm{val}}^{+}\cup E_{\mathrm{test}}^{+}\right)$$ (11)

$$E_{\mathrm{aug}}=E_{\mathrm{train}}^{+}\cup E_{\mathrm{prior}}^{*}$$ (12)

$$A_{\mathrm{aug}}\left[i,j\right]=1\text{ if }\left(i,j\right)\in E_{\mathrm{aug}}$$ (13)

where $E_{\mathrm{prior}}$ represents the external prior interactions, $E_{\mathrm{train}}^{+}$ denotes the set of positive training TF–target interactions, $E_{\mathrm{val}}^{+}$ and $E_{\mathrm{test}}^{+}$ denote the validation and test positive interaction sets, respectively. The final augmented edge set $E_{\mathrm{aug}}$ is constructed by combining the training interactions with the filtered prior edges. The adjacency matrix $A_{\mathrm{aug}}$ is defined such that $A_{\mathrm{aug}}\left[i,j\right]=1$ if there exists a directed interaction from gene i to gene j , and 0 otherwise. Figure 1 presents the MultiCausGRN input, output, and workflow.

### 2.2 Datasets

The study used publicly available datasets comprising scRNA-ATAC-seq data from different tissues in humans and mice. Table 1 shows the details of the datasets used in this study. The BEELINE framework provides several benchmark datasets for GRN inference; however, most of the original BEELINE datasets are based solely on single-cell RNA sequencing data and do not include paired chromatin accessibility measurements. Since MultiCausGRN is designed to integrate both gene expression and chromatin accessibility information, we selected the VSC, mCAD, and HSC benchmark subsets, which provide compatible multi-omics regulatory benchmark graphs. These benchmark datasets are intentionally constructed as small curated regulatory networks for controlled evaluation and therefore contain only a limited number of genes and experimentally validated regulatory interactions (5–11 genes and 14–30 positive edges after benchmark construction), despite originating from datasets containing approximately 2,000 cells. Consequently, they serve as standardized benchmark scenarios for validating model behavior under controlled conditions. To evaluate performance in a more realistic large-scale multi-omics setting, we additionally employed the PBMC dataset containing 10,970 cells, 3,718 genes, and a substantially larger regulatory search space (Swanson et al., 2021; Wehrkamp-Richter, 2022). Additionally, in the field of GRN, there has been a persistent lack of real-world networks for model evaluation. In the GRN inference literature, a common practice is to evaluate the accuracy of a resulting network by comparing its edges to an appropriate database of TFs and their targets. Therefore, we utilized the curated TF–target regulatory interactions from OmniPath (Türei et al., 2016) and the functional interactions from the UniProt (UniProt Consortium, 2021) database as ground truth networks.

**TABLE 1:** Statistics of scRNA-ATAC-seq datasets with the ground-truth networks composed of TFs and variable genes from the BEELINE dataset and top 5,000 most variable genes from the PBMC dataset.

Dataset	Organism	Tissue type	Original cells	Genes	TFs	Targets	Positive edges	Network density
VSC	Mouse	Vascular smooth muscle	2000	8	8	8	15	0.234
mCAD	Mouse	Coronary artery disease model	2000	5	5	5	14	0.560
HSC	Mouse	Hematopoietic stem cells	2000	11	11	11	30	0.248
PBMC	Human	Peripheral blood mononuclear cells	10,970	3,718	24	168	224	—

The BEELINE datasets represent controlled benchmark scenarios with predefined regulatory sparsity levels, whereas the PBMC dataset reflects a realistic, large-scale regulatory inference setting.

#### 2.2.1 BEELINE datasets

We conducted experiments on datasets from the BEELINE benchmarking platform (Pratapa et al., 2020). BEELINE provides standardized datasets and curated gold-standard regulatory networks for systematic comparison of GRN inference methods. In this study, we focused on three multi-omics datasets from the BEELINE benchmark, namely: i)VSC, ii)mCAD, and iii)HSC. These datasets contain paired single-cell measurements of gene expression and chromatin accessibility, enabling the integration of transcriptional and regulatory information for GRN reconstruction. Across all datasets, regulatory networks are represented as directed graphs, where nodes correspond to genes and edges represent transcription factor (TF) regulatory interactions. The VSC dataset comprises single-cell multi-omics measurements from vascular cell populations. The dataset includes both scRNA-seq expression profiles and chromatin accessibility signals obtained through scATAC-seq. These complementary modalities provide insights into both transcriptional activity and regulatory potential. Following standard preprocessing procedures, low-quality cells and low-expressed genes were filtered out, and highly variable genes were retained for downstream analysis. Chromatin accessibility signals associated with promoter and regulatory regions were aggregated at the gene level and used as additional regulatory features. This multi-omics representation allows more accurate modelling of transcription factor activity and regulatory relationships. The mCAD dataset represents a multi-omics single-cell dataset capturing transcriptional regulation during mouse cardiac development. It provides simultaneous measurements of gene expression and chromatin accessibility across multiple cellular states involved in cardiovascular differentiation. Due to the dynamic regulatory processes underlying cardiac development, the dataset exhibits substantial cellular heterogeneity and complex regulatory patterns. These properties make it a challenging benchmark for GRN inference methods. For this dataset, gene expression matrices were normalized and filtered to retain highly informative genes. Chromatin accessibility signals were integrated with expression features to capture regulatory cues associated with transcription factor binding. The HSC dataset contains single-cell multi-omics profiles of hematopoietic stem cells. This dataset captures regulatory programs governing early hematopoietic differentiation and lineage commitment. Hematopoietic systems are characterized by hierarchical regulatory networks involving multiple transcription factors controlling lineage specification. Consequently, the HSC dataset represents a biologically complex setting for GRN inference. Expression and chromatin accessibility data were integrated to construct a multi-omics feature representation for each gene. This joint representation provides additional regulatory context beyond gene expression alone.

#### 2.2.2 PBMC multi-omics dataset

We evaluated the proposed model on a peripheral blood mononuclear cell (PBMC) multi-omics dataset, which integrates gene expression and chromatin accessibility measurements at single-cell resolution. This dataset was generated using single-cell multi-modal sequencing technologies, enabling simultaneous profiling of: i)Gene expression (scRNA-seq) and ii)Chromatin accessibility (scATAC-seq). The PBMC dataset contains thousands of immune cells representing heterogeneous cell populations, including T cells, B cells, and monocytes. This cellular diversity provides a realistic and challenging setting for GRN inference.

The raw expression matrix was preprocessed as follows: i)Filtering low-quality cells and low-expressed genes. ii)Log-normalization of expression counts. iii)Selection of highly variable genes. v)Alignment of gene identifiers across omics modalities. After preprocessing, the final expression matrix consisted of approximately 3,000–5,000 genes across more than ten thousand cells, forming the node feature matrix used in the MultiCausGRN model.

To construct gold-standard regulatory interactions for supervised learning, curated transcription factor–target relationships were collected from the publicly available regulatory database OmniPath. OmniPath integrates literature-curated signaling and transcriptional regulatory interactions from multiple pathway resources, providing a comprehensive collection of experimentally supported TF–target relationships. In this study, regulatory interactions were first mapped from UniProt protein identifiers to gene symbols using UniProt annotation tables. Subsequently, only TF–target pairs corresponding to genes present in the processed single-cell expression matrix were retained. These filtered interactions were used to generate positive regulatory edges, while negative samples were created by randomly pairing transcription factors with non-target genes. The resulting dataset was split into training, validation, and testing subsets for model evaluation.

Multi-omics features or chromatin accessibility profiles, were integrated with gene expression features to provide additional regulatory context. Accessibility signals corresponding to promoter and enhancer regions were aggregated at the gene level and concatenated with expression-derived features to construct the final multi-omics feature representation. This integration enables the proposed model to capture both transcriptional activity and regulatory potential, thereby improving the robustness of GRN inference.

### 2.3 Data pre-processing

Raw gene-expression matrices were first standardized to a unified format in which rows correspond to genes and columns to single cells. For each dataset, genes with extremely low expression across cells were removed to reduce noise and computational complexity. Highly variable gene (HVG) selection was then performed to retain the most informative transcriptional features for regulatory network inference. Specifically, the top 5,000 highly variable genes were selected for the PBMC dataset to reduce noise, improve computational tractability, and retain the most informative genes for regulatory inference. 3,718 genes were retained for the PBMC multi-omics dataset after quality filtering. These genes were used as the input feature space for model representation learning. Gene expression values were standardized using z-score normalization (StandardScaler) before graph learning. The supervised TF–target prediction task was then defined using high-confidence regulatory interactions from OmniPath. Among the filtered genes, 153 transcription factors were identified. To construct the supervised benchmark network, curated regulatory interactions from OmniPath were mapped onto the processed PBMC gene set. Only TF-target interactions for which both genes were present in the processed feature space were retained. This filtering resulted in a high-confidence benchmark network comprising 24 transcription factors, 168 target genes, and 224 positive regulatory interactions. Only positive training edges were used to build the graph adjacency. Validation and test interactions were excluded from graph construction. Importantly, the full set of 3,718 genes was retained for feature construction and graph representation learning, while the 224 curated interactions were used solely as supervision and evaluation labels. Therefore, the final supervised network is intentionally smaller than the original gene set, but it provides a biologically grounded benchmark for assessing whether MultiCausGRN can recover known regulatory relationships from paired scRNA-seq and scATAC-seq features. Gene identifiers were harmonized across expression matrices and prior regulatory resources to ensure consistent mapping between transcription factors and target genes. In particular, gene name prefixes introduced during preprocessing were removed, and duplicate mappings were resolved by retaining the first occurrence. A candidate regulatory space was subsequently constructed by defining a set of transcription factors and potential target genes based on curated prior knowledge resources. Positive regulatory edges were obtained from the prior regulatory network and aligned to the filtered gene expression matrix. To construct supervised learning datasets for GRN inference, negative TF–target pairs were generated using uniform random sampling from the remaining candidate TF–target combinations while ensuring no overlap with known positive interactions. Negative interactions were generated by randomly sampling TF–target pairs that were not present in the benchmark positive regulatory network. These sampled interactions were treated as negative examples during training and evaluation. As is common in GRN benchmarking, some sampled negatives may correspond to currently uncharacterized or undiscovered regulatory interactions rather than confirmed biological negatives. Consequently, the reported performance metrics should be interpreted within the limitations of available regulatory knowledge. The final labeled interaction dataset was randomly divided into training (70%), validation (15%), and testing (15%) subsets. Finally, the processed gene expression matrix together with TF indices, target indices, and labeled interaction splits were formatted as input for the MultiCausGRN model.

### 2.4 Prior-guided regulatory modeling

**FIGURE 2:**
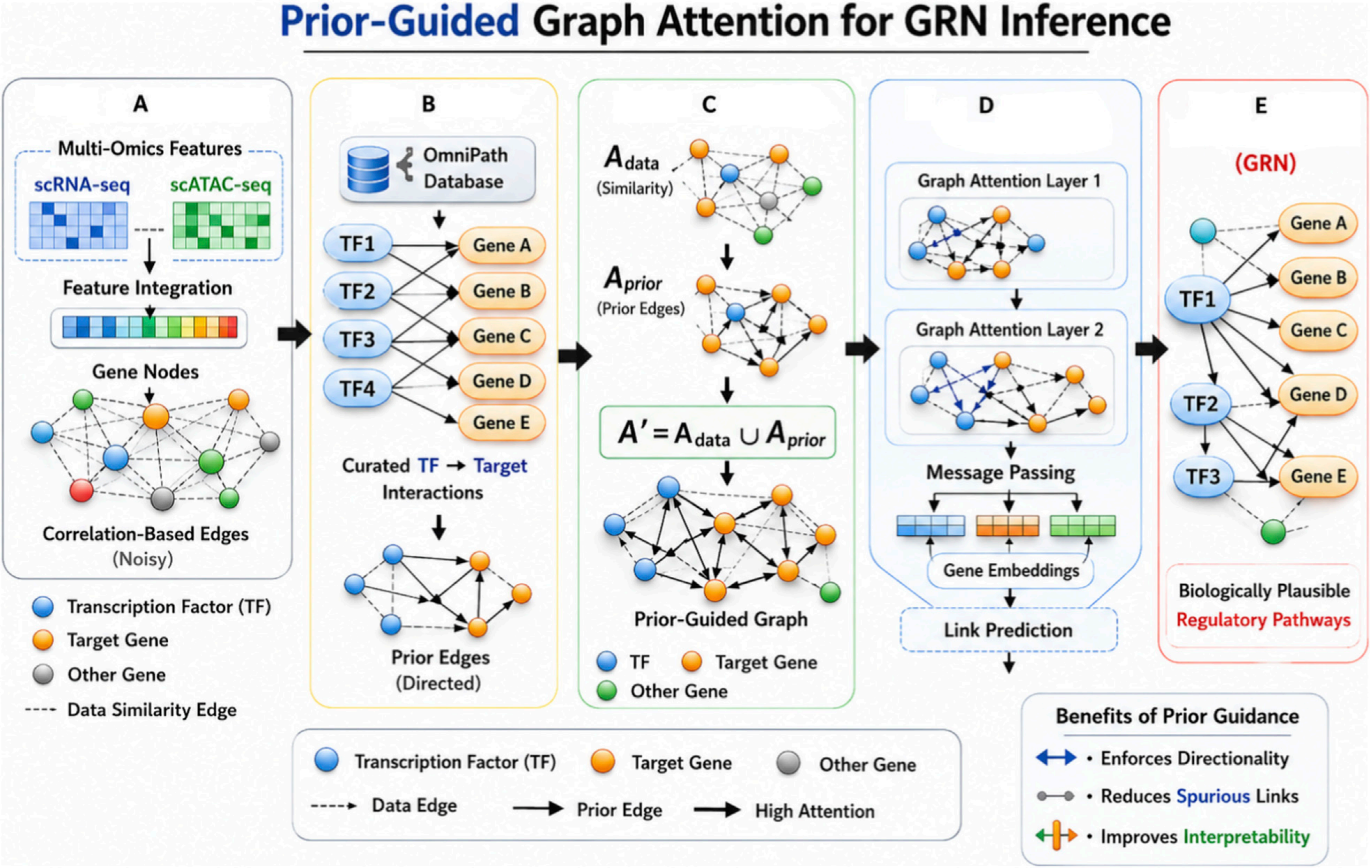
Data-driven similarity edges derived from multi-omics features for MultiCausGRN Model. A curated prior network of transcription factor–target interactions is incorporated to augment the graph structure. During graph attention learning, prior edges bias information propagation toward biologically plausible regulatory directions, resulting in more accurate and interpretable gene regulatory network predictions. **(A)** Data-Driven Graph. **(B)** Prior Network. **(C)** Augmented Graph Multi-Omics Features. **(D)** Graph Attention Learning (GAT). **(E)** Inferred Gene Regulatory Network.

To incorporate biologically grounded regulatory directionality, MultiCausGRN integrates a curated prior transcription factor–target interaction network into the graph learning process. A directed prior graph $G_{p}=\left(V,E_{p}\right)$ is constructed using experimentally validated regulatory interactions obtained from the OmniPath and TRRUST (Han et al., 2015) databases. Only interactions corresponding to genes present in the analyzed multi-omics training dataset are retained. The prior regulatory graph is then used to augment the data-driven adjacency matrix. Specifically, the final graph used for message passing is defined as the union of similarity-based edges derived from multi-omics features and directed prior edges representing putative regulatory relationships. This augmentation introduces directional constraints that bias information propagation toward biologically plausible transcription factor–target pathways. During graph attention learning, node embeddings are updated using neighborhood aggregation over the augmented graph. Consequently, the attention mechanism assigns higher importance to edges supported by prior regulatory knowledge, enabling the model to emphasize mechanistically relevant interactions while suppressing spurious correlations arising from noisy single-cell measurements. Unlike approaches that rely on large external reference datasets or motif-constrained post-processing, the proposed model incorporates prior knowledge directly within the representation learning stage. This design improves both predictive robustness and biological interpretability, as demonstrated in downstream GRN reconstruction experiments. Figure 2 represents how the prior edges approach is employed in the MultiCausGRN Model flow.

### 2.5 MultiCausGRN implementation details

MultiCausGRN was implemented in PyTorch and trained using the Adam optimizer with an initial learning rate of 0.003. The model was trained for 90 epochs with a batch size of 256. A StepLR learning-rate scheduler was employed with a decay factor of 0.99 applied after each epoch. Model selection was performed using the validation AUPRC, and the checkpoint achieving the highest validation AUPRC was used for final testing. The graph encoder consists of two GAT layers. The first GAT layer employs three attention heads with 128 hidden units per head, while the second GAT layer employs three attention heads with 64 hidden units per head. Outputs from the first layer are concatenated and passed through an ELU activation function before being processed by the second GAT layer. The attention mechanism uses a LeakyReLU activation with a negative slope coefficient of 0.2. The resulting graph representations are projected into separate TFs and target gene embedding spaces through two independent feed-forward networks. Each projection network consists of two fully connected layers with dimensions 64,32, and 16. A dropout rate of 0.01 is applied after the first projection layer to reduce overfitting. Regulatory interaction scores are computed using a dot-product decoder between transcription factor and target gene embeddings, followed by a sigmoid activation for binary interaction prediction. Gene expression matrices were standardized using z-score normalization (StandardScaler) before graph learning. Graph adjacency matrices were constructed exclusively from positive regulatory interactions. For the baseline configuration, only positive interactions from the training split were used to construct the graph structure, thereby preventing information leakage from validation and test interactions. For prior-knowledge experiments, the adjacency matrix was constructed from curated prior regulatory interactions after removing any edges overlapping with validation and test positive interactions. All experiments were conducted using five independent random seeds (1, 2, 4, 8, and 16). Random seeds were applied consistently to NumPy, PyTorch, and data sampling procedures to ensure reproducibility. The PBMC multi-omics dataset was obtained from the 10x Genomics Chromium Single Cell Multiome platform, which simultaneously profiles gene expression and chromatin accessibility from the same cell (Wehrkamp-Richter, 2022). The final reported results correspond to the mean and standard deviation across the five independent runs. Detailed preprocessing and hyperparameter settings are reported in Supplementary Tables S1, S2.

### 2.6 Evaluation strategies

The performance of the proposed model was evaluated using curated transcription factor–target regulatory networks as ground truth. Due to the inherent sparsity of gene regulatory networks and the large imbalance between observed and unobserved regulatory interactions, a supervised link prediction evaluation protocol was adopted. For each dataset, known TF–target regulatory pairs were considered as positive samples. These interactions were randomly divided into training (70%), validation (15%), and test (15%) subsets. To construct negative samples, transcription factors were randomly paired with genes that were not reported as regulatory targets in the gold-standard network. Considering the extremely large number of potential negative TF–gene combinations, a uniform random negative sampling strategy was applied to generate a balanced set of negative samples. Specifically, the number of negative pairs was matched to the number of positive pairs to mitigate class imbalance during training. To enhance the discriminative ability of the model, hard negative sampling was employed in the training phase. For each positive TF–target pair $\left(T_{a},G_{b}\right)$ , a corresponding negative pair $\left(T_{a},G_{c}\right)$ sharing the same transcription factor was sampled. This strategy forces the model to distinguish between true and false targets of the same regulator, which has been shown to improve representation learning and convergence stability. To prevent information leakage, negative samples used in the test set were drawn exclusively from the remaining unobserved TF–gene interactions not used during training or validation. The ratio of positive to negative samples in the test set was maintained consistent with that in the overall constructed dataset.

Model performance was evaluated using standard link prediction metrics, including Area Under the Receiver Operating Characteristic Curve (AUROC), and Area Under the Precision–Recall Curve (AUPRC). Since gene regulatory networks are highly sparse, AUPRC was considered the primary evaluation metric, as it better reflects performance under severe class imbalance. To ensure robustness and reproducibility, all experiments were repeated with multiple random seeds, and the mean performance and standard deviation were reported. In addition to predictive accuracy, we further assessed the biological plausibility of the inferred regulatory networks through: Precision at Top-K predicted edges, PCA analysis, and network topology characteristics, such as the transcription factor degree distribution. These analyses provide complementary evidence for the effectiveness of incorporating prior regulatory knowledge into the model.

## 3 Results

### 3.1 Performance of MultiCausGRN on benchmark BEELINE multi-omics datasets

**FIGURE 3:**
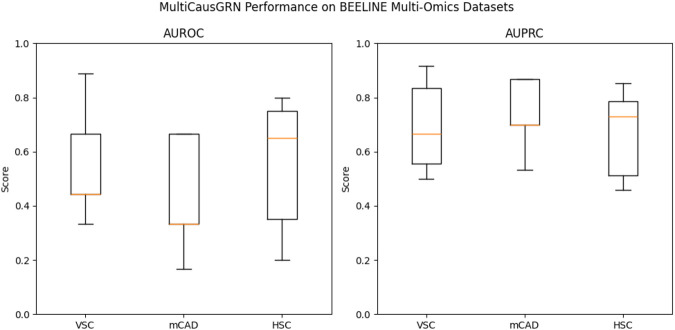
Performance of MultiCausGRN on BEELINE multi-omics benchmark datasets. Mean test AUROC and AUPRC values across five random seeds are shown for VSC, mCAD, and HSC. Error bars represent the standard deviation across runs. The observed variability reflects the limited size of the benchmark regulatory graphs, which contain only 14–30 experimentally validated regulatory interactions.

To evaluate the generalizability of MultiCausGRN, we first assessed the model on three multi-omics benchmark datasets obtained from the BEELINE framework: VSC, mCAD, and HSC. These datasets correspond to curated benchmark regulatory graphs designed for controlled evaluation of GRN inference methods. Unlike the larger PBMC dataset, the benchmark graphs contain only 14–30 experimentally validated regulatory interactions and therefore represent substantially smaller regulatory inference tasks. To account for variability arising from the limited number of regulatory interactions, each experiment was repeated using five random seeds, and the mean and standard deviation of AUROC and AUPRC were reported. Table 2 summarizes the performance of MultiCausGRN on the BEELINE benchmark datasets. The model achieved mean AUROC values of 0.556, 0.433, and 0.550 on VSC, mCAD, and HSC, respectively. Corresponding AUPRC values were 0.694, 0.733, and 0.668. Although performance varied across datasets, MultiCausGRN consistently recovered known TF–target interactions from paired gene expression and chromatin accessibility data. The relatively large standard deviations observed across seeds are expected, given the limited size of the benchmark graphs. For example, the VSC and mCAD datasets contain only 15 and 14 positive regulatory interactions, respectively, while HSC contains 30 positive interactions. Under these conditions, a small number of edge-level prediction changes can substantially affect AUROC and AUPRC values. Consequently, these benchmark datasets primarily serve as controlled validation scenarios rather than comprehensive large-scale evaluations. Overall, the BEELINE experiments demonstrate that MultiCausGRN can identify biologically meaningful regulatory relationships across multiple benchmark networks. However, due to the limited size of these benchmark graphs, the PBMC dataset was used as the primary benchmark for evaluating robustness, scalability, and biological relevance in a realistic multi-omics setting. Performance of MultiCausGRN on BEELINE multi-omics datasets is presented in Figure 3.

**TABLE 2:** Performance of MultiCausGRN on BEELINE multi-omics benchmark datasets.

Dataset (BEELINE)	Test AUROC	Test AUPRC
VSC	0.556 ± 0.227	0.694 ± 0.175
mCAD	0.433 ± 0.224	0.733 ± 0.144
HSC	0.550 ± 0.281	0.668 ± 0.171

Results are reported as mean ± standard deviation across five random seeds. The benchmark graphs contain between 14 and 30 experimentally validated regulatory interactions and provide controlled evaluation scenarios for multi-omics GRN inference.

### 3.2 Performance of MultiCausGRN on and PBMC multi-omics datasets

**FIGURE 4:**
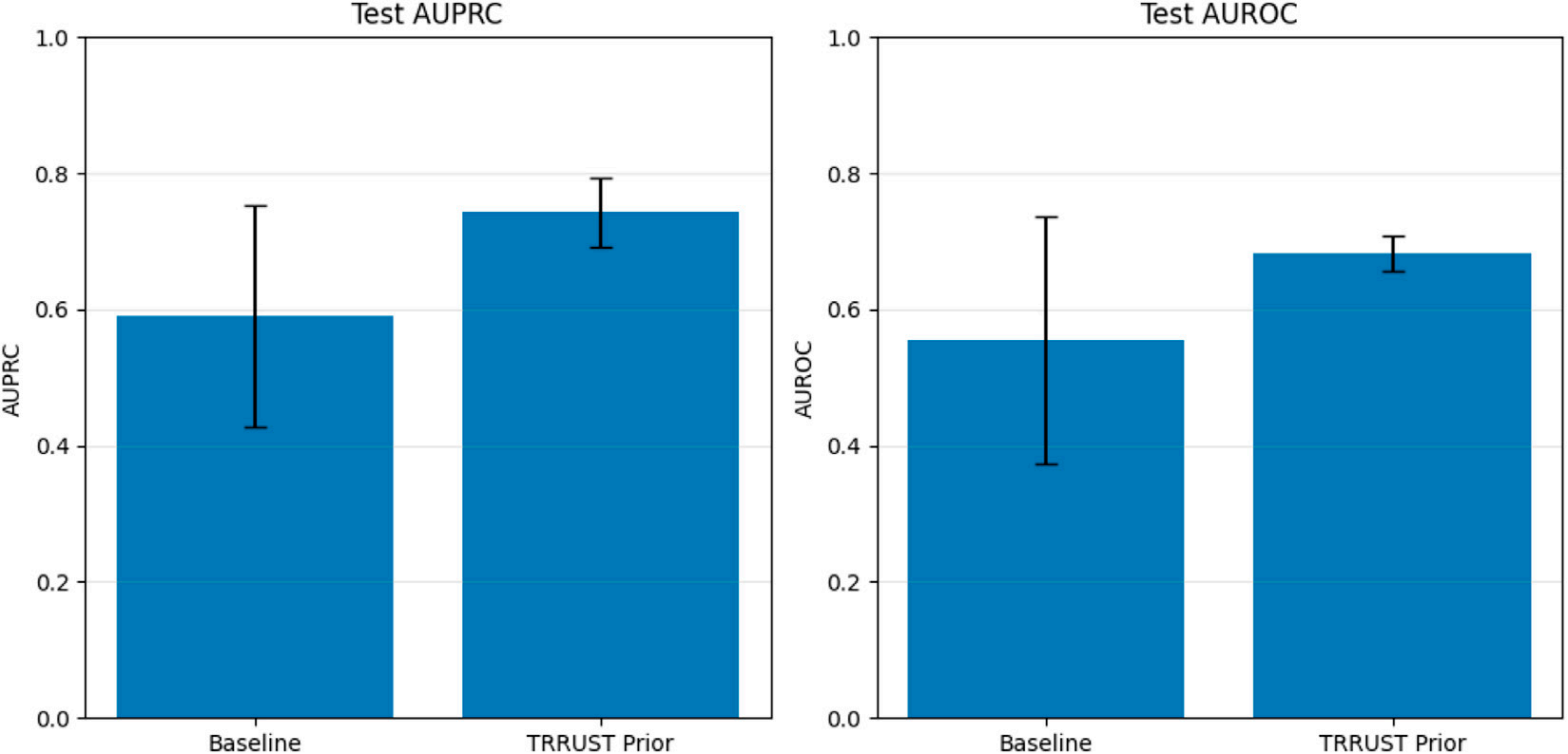
Overall performance of MultiCausGRN across the PBMC multi-omics dataset. The integration of prior information further improves predictive performance compared with the baseline model without prior knowledge.

We evaluated the model on the more challenging PBMC multi-omics dataset, which represents a realistic regulatory inference scenario. In this setting, we compared the standard multi-omics configuration against the prior-guided variant across five random seeds. Without prior guidance, the model achieved a mean validation AUPRC of 0.804 ± 0.014, a mean test AUROC of 0.554 ± 0.182, and a mean test AUPRC of 0.590 ± 0.163 across five seeds. After incorporating prior-guided information, the mean validation AUPRC was 0.85 ± 0.0150, the mean test AUROC increased to 0.682 ± 0.026, and the mean test AUPRC increased to 0.743 ± 0.049. Across five random seeds, the best-performing prior-guided run achieved a test AUROC of 0.712 and test AUPRC of 0.782. These results suggest that the prior knowledge did not substantially change validation ranking performance, but it improved test-set generalization and reduced variability across random seeds. In particular, the prior knowledge variant yielded higher average test AUROC and AUPRC and produced more consistent performance across repeated runs. This indicates that prior-guided regulatory structure can enhance robustness in realistic multi-omics GRN inference tasks. Table 3 and Figure 4 present the results of the MultiCausGRN model for the PBMC datasets.

**TABLE 3:** Performance of the MultiCausGRN model on the PBMC datasets.

Configuration	Prior source	Prior edges	Val AUPRC	Test AUROC	Test AUPRC
Baseline	Train-positive adjacency	152	0.804 ± 0.014	0.554 ± 0.182	0.590 ± 0.163
TRRUST-guided	Filtered TRRUST	36	**0.85** ± **0.0150**	**0.682** ± **0.026**	**0.743** ± **0.049**

The baseline model without prior knowledge exhibited high variability, with a test AUPRC of 0.590 ± 0.163 and AUROC of 0.554 ± 0.182, indicating unstable learning behavior. In contrast, incorporating the TRRUST prior significantly improved both performance and stability, achieving a test AUPRC of 0.743 ± 0.049 and AUROC of 0.682 ± 0.026. Bold values represent the highest value for each evaluation metric.

**FIGURE 5:**
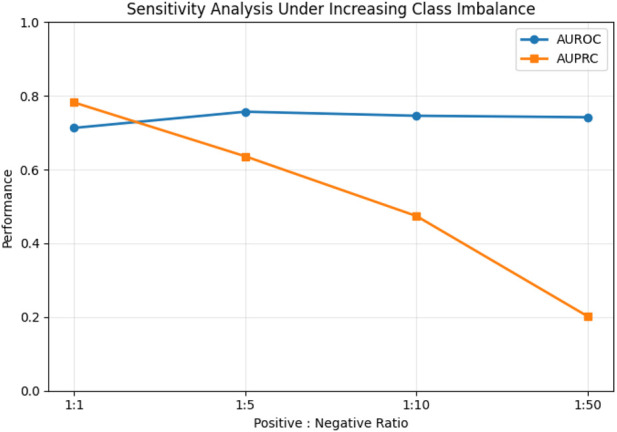
Sensitivity analysis of MultiCausGRN under increasing class imbalance. The model was evaluated using the same trained configuration (seed = 2) while progressively increasing the number of negative TF–target interactions in the test set. AUROC remained relatively stable across all evaluation settings, whereas AUPRC decreased as the class imbalance increased. This behavior is expected in sparse GRN reconstruction tasks and reflects the increasing difficulty of recovering true regulatory interactions within a substantially larger candidate interaction space.

To assess the robustness of MultiCausGRN under more realistic GRN sparsity conditions, we performed an additional sensitivity analysis using increasingly imbalanced test distributions. While the primary benchmark employed a balanced positive-to-negative ratio (1:1), additional evaluations were conducted using 1:5, 1:10, and 1:50 ratios while keeping the trained model configuration fixed (seed = 2). As shown in Figure 5, AUROC remained relatively stable across all evaluation settings, ranging from 0.713 to 0.757, indicating that the model consistently ranked true regulatory interactions above randomly sampled negatives. In contrast, AUPRC decreased from 0.782 under the balanced setting to 0.636, 0.474, and 0.202 for the 1:5, 1:10, and 1:50 settings, respectively. This reduction is expected because precision-recall metrics are highly sensitive to class imbalance and become increasingly challenging as the number of candidate interactions grows. Despite the decrease in AUPRC, MultiCausGRN maintained meaningful discriminative performance even under the highly sparse 1:50 setting. These findings suggest that the proposed framework remains robust under more realistic GRN reconstruction scenarios and that the strong performance observed in the balanced benchmark is not solely a consequence of the evaluation protocol. Table 4 concludes the performance of MultiCausGRN across all ratios. The number of positive interactions was maintained at 32, whereas the number of negative interactions was progressively increased from 32 to 1,600.

**TABLE 4:** Sensitivity analysis of MultiCausGRN under increasingly imbalanced evaluation settings.

Ratio	Positives	Negatives	AUROC	AUPRC
1:1	32	32	0.713	0.782
1:5	32	160	0.757	0.636
1:10	32	320	0.746	0.474
1:50	32	1,600	0.742	0.202

The number of positive interactions was fixed (n = 32), while the number of negative interactions was progressively increased to simulate realistic GRN sparsity.

To further assess statistical uncertainty associated with the relatively small benchmark size, bootstrap resampling was performed on the primary balanced PBMC test set. MultiCausGRN achieved an AUROC of 0.712 (95% CI: 0.574–0.837) and an AUPRC of 0.782 (95% CI: 0.643–0.887). These confidence intervals indicate that the model maintains predictive performance across repeated resampling of the test interactions. Together with the multi-seed evaluation and class-imbalance experiments, these results provide additional evidence for the robustness of the proposed framework. Supplementary Tables S3, S4 present the results of multi-seed evaluation and bootstrap confidence intervals. One limitation of the current evaluation framework is the construction of negative interactions through random sampling of TF–target pairs not present in the benchmark regulatory network. Although this strategy is widely used in GRN inference studies, the absence of an interaction from current databases does not guarantee the absence of biological regulation. Therefore, some sampled negatives may correspond to undiscovered regulatory relationships, potentially leading to an underestimation of model performance. Additional implementation details, preprocessing parameters, bootstrap confidence intervals, and per-seed performance results are provided in the Supplementary Material.

### 3.3 Effect of prior edges integration

**FIGURE 6:**
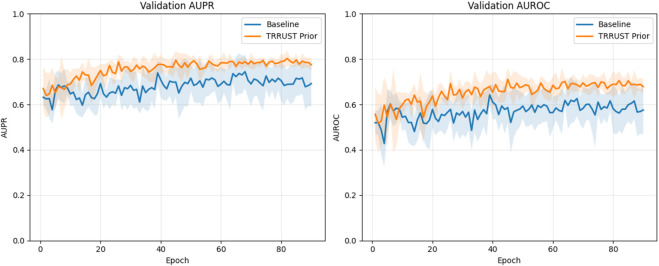
Robustness analysis of the MultiCausGRN across different random seeds on the PBMC dataset. Test AUROC and AUPRC values are shown for five independent training runs. The model with prior knowledge demonstrates improved consistency and reduced performance variance compared with the baseline model, suggesting enhanced training stability when incorporating biologically informed prior edges.

**FIGURE 7:**
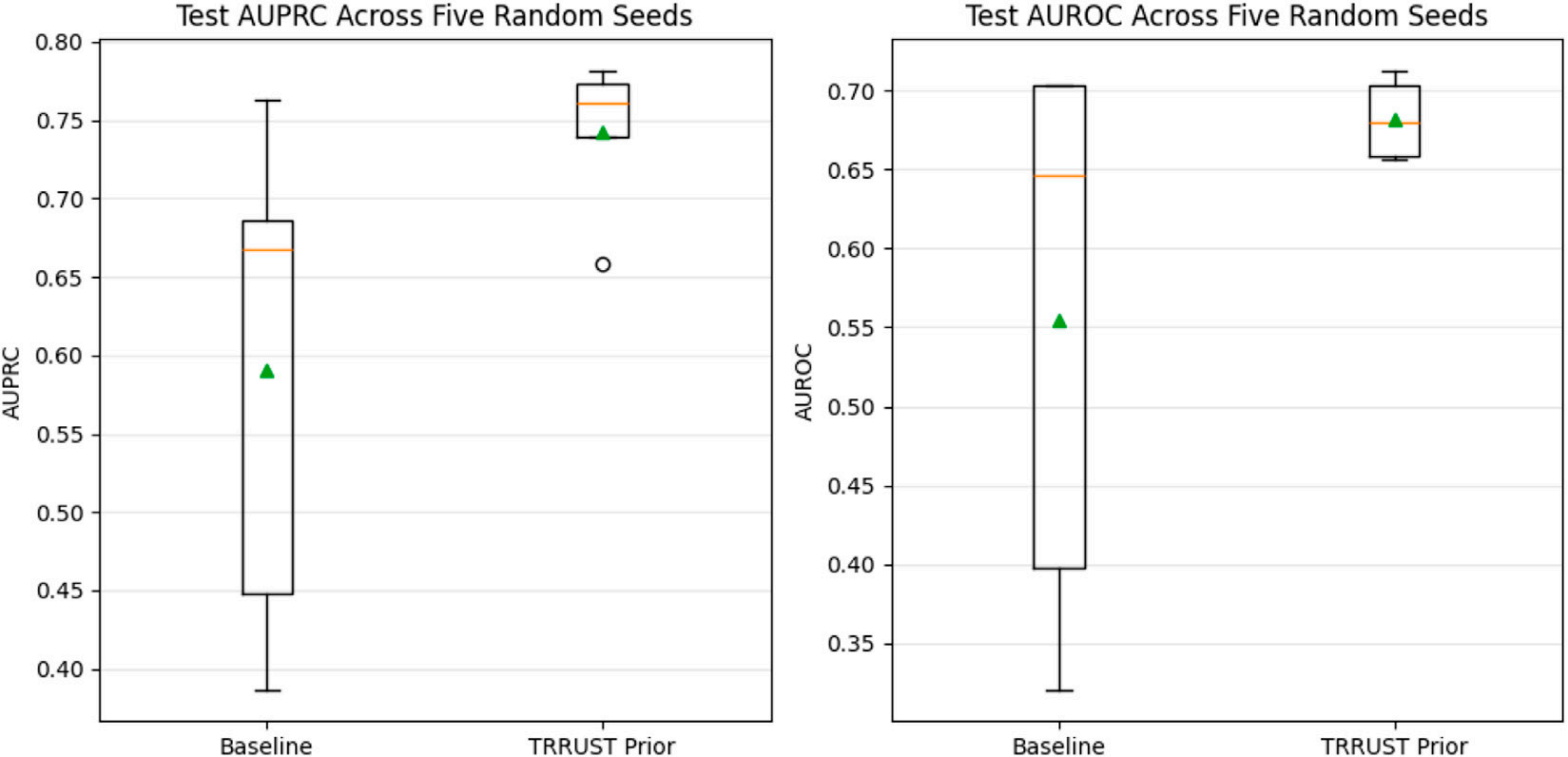
Stability analysis of MultiCausGRN across five random seeds. The baseline model constructs the message-passing graph using training positive interactions only, whereas the TRRUST-guided model incorporates an external curated regulatory prior. Boxplots show the distribution of AUPRC and AUROC across five independent runs. The TRRUST prior improves both predictive performance and robustness, resulting in higher median scores and reduced variance across seeds.

To evaluate the contribution of biologically informed prior knowledge, we incorporated experimentally supported transcription factor–target interactions into the MultiCausGRN model as structural constraints during graph construction. The prior edges were derived from the OmniPath and TRRUST v1 (Han et al., 2015) databases and integrated into the model by augmenting the adjacency matrix used for graph attention learning. The baseline message-passing graph was constructed using only positive regulatory interactions from the training split. Validation and test-positive interactions were not included in the adjacency graph. After leakage filtering, the internal OmniPath prior contained 152 edges, corresponding exactly to the training positive interactions. Therefore, it was equivalent to the baseline train-positive adjacency and was not treated as an independent prior source. For the external prior setting, TRRUST regulatory interactions were mapped to the PBMC gene set and filtered to remove validation and test positive interactions, yielding 36 prior edges. Consequently, the model was required to generalize to previously unseen TF–target pairs during evaluation rather than recovering edges already present in the graph structure. Importantly, the adjacency matrix was used only to define the neighborhood message-passing structure during representation learning and did not directly expose validation or test labels to the prediction objective. Performance was assessed using AUROC and AUPRC across five independent runs with different random seeds to evaluate both accuracy and robustness. The baseline model exhibited unstable performance across random seeds, with test AUPRC ranging from 0.38 to 0.76. This variability indicates that the model struggles to learn meaningful regulatory structure in the absence of additional prior knowledge. In contrast, incorporation of the external TRRUST prior improved both predictive performance and stability, achieving a test AUROC of 0.682 ± 0.026 and a test AUPRC of 0.743 ± 0.049. Notably, the TRRUST prior contained only 36 regulatory interactions yet consistently outperformed the baseline configuration. These findings suggest that high-confidence biological regulatory knowledge provides more informative guidance for graph attention learning than relying solely on training-derived interactions. Although the performance gain in AUROC was moderate, the improvement in AUPRC and reduced variance highlight the effectiveness of prior knowledge constraints in learning transcriptional regulatory relationships from noisy multi-omics data. Figure 6 shows the AUROC and AUPRC for the model training with and without prior knowledge integration. Figures 4–7, illustrate the overall performance of MultiCausGRN model.

### 3.4 Biological validation of the inferred GRN

#### 3.4.1 Principal component analysis (PCA) analysis

**FIGURE 8:**
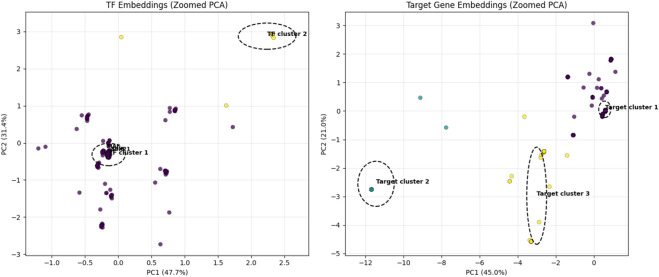
Principal component analysis (PCA) visualization of learned TF and target gene embeddings. PCA was computed using all learned embeddings, while extreme outliers were excluded from the displayed view to improve cluster visibility. Dashed ellipses indicate embedding clusters identified by K-means, and selected regulatory genes are labeled.

To investigate whether MultiCausGRN learns biologically meaningful latent representations, we visualized the learned TFs and target gene embeddings using principal component analysis (PCA). Figure 8 shows the PCA projections of the embeddings generated by the TRRUST-guided MultiCausGRN model. PCA was performed on the complete embedding space, while extreme outliers were excluded from the displayed visualization to improve cluster visibility. For the TF embeddings, the first two principal components explain 47.7% and 31.4% of the total variance, respectively, accounting for approximately 79.1% of the embedding variation. The TF embeddings form two clearly distinguishable clusters, suggesting that the model learns distinct regulatory representations for groups of TFs with potentially different regulatory roles. In addition, several TFs, including GATA3, appear as isolated outliers, indicating unique regulatory characteristics that differ substantially from the dominant TF groups. The target gene embeddings also exhibit structured organization. The first two principal components explain 45.0% and 21.0% of the variance, respectively, accounting for approximately 66.0% of the total variation. Three major clusters can be observed, indicating that target genes with similar regulatory contexts are mapped to nearby regions in the latent space. Compared with TF embeddings, the target embeddings display greater dispersion, reflecting the diverse and overlapping regulatory programs associated with downstream genes. The observed clustering patterns suggest that MultiCausGRN learns informative low-dimensional representations that preserve biologically relevant regulatory structure. The separation of TF and target gene groups, together with the presence of distinct hub-gene outliers, indicates that integrating multi-omics information and curated regulatory priors enables the model to capture meaningful regulatory relationships beyond simple expression similarity. These findings provide qualitative evidence that the learned embeddings encode regulatory information that supports accurate gene regulatory network inference.

#### 3.4.2 Top hub TFs analysis

**FIGURE 9:**
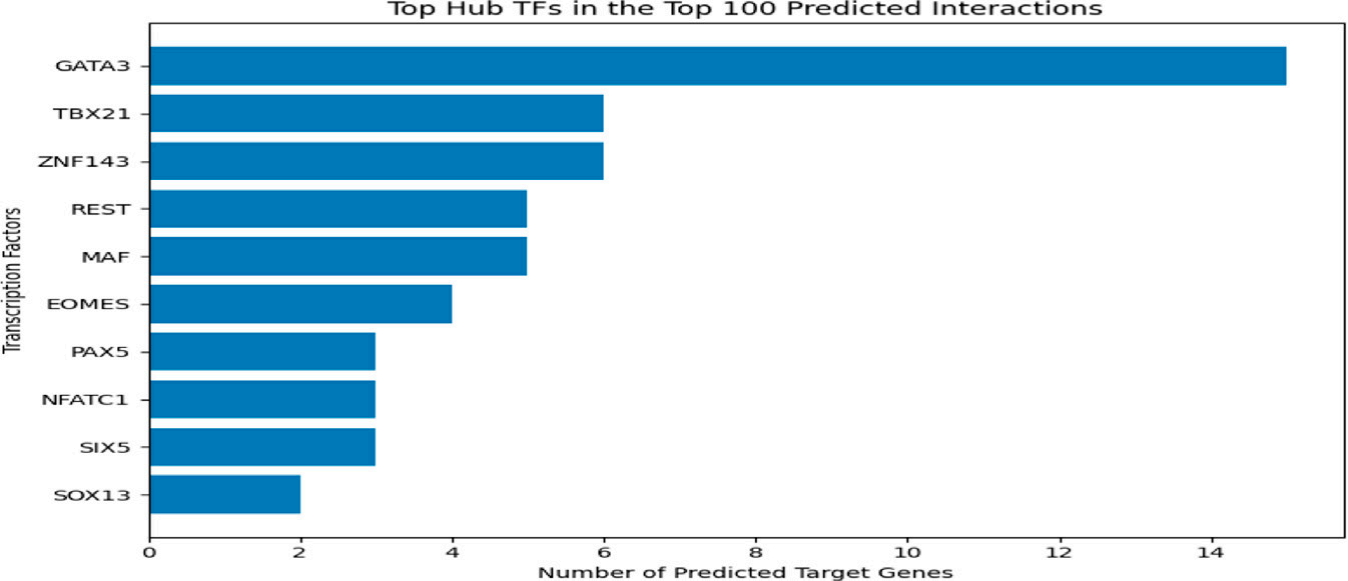
Top hub transcription factors identified from the top 100 predicted regulatory interactions generated by MultiCausGRN under the TRRUST prior configuration. TFs are ranked according to the number of predicted target genes. Several biologically relevant immune-associated regulators, including GATA3, TBX21, BATF, and NFATC1, were identified as major hubs.

Figure 9 presents the TFs most frequently represented among the highest-confidence predicted regulatory interactions inferred by MultiCausGRN. GATA3 emerged as the dominant transcriptional hub, exhibiting the largest number of predicted target interactions. Additional highly ranked regulators included TBX21, MAF, EOMES, NFATC1, PAX5, REST, and ZNF143. Several of these transcription factors are well-established regulators of immune-cell differentiation, cytokine signaling, and lymphocyte development. In particular, GATA3 and TBX21 are key regulators of T-helper cell lineage specification, whereas NFATC1, MAF, and EOMES participate in immune activation and differentiation programs (Lazarevic et al., 2013; Zhu, 2010). The recovery of these biologically relevant regulators supports the plausibility of the inferred PBMC regulatory network and suggests that MultiCausGRN captures meaningful regulatory programs rather than merely learning statistical associations from the input data.

#### 3.4.3 Functional enrichment analysis

**FIGURE 10:**
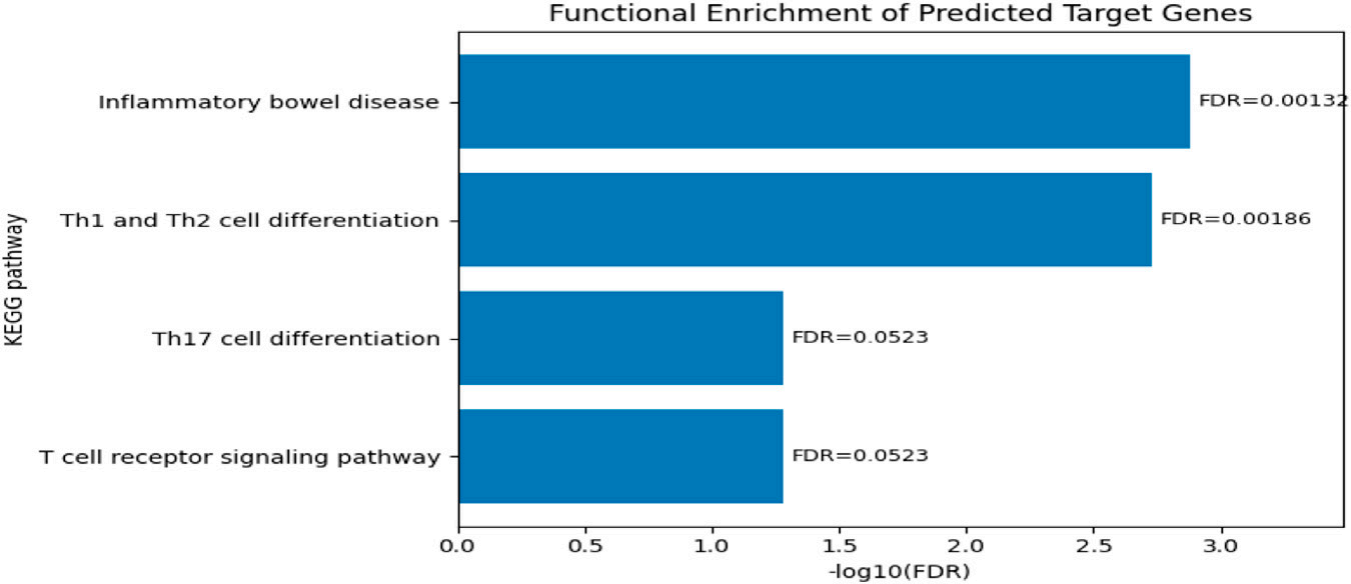
Functional enrichment analysis of the highest-confidence target genes predicted by MultiCausGRN. KEGG enrichment revealed immune-related pathways, including inflammatory bowel disease, Th1 and Th2 cell differentiation, Th17 cell differentiation, and T-cell receptor signaling, supporting the biological relevance of the inferred PBMC regulatory network.

To assess the biological relevance of the inferred GRN, we performed functional enrichment analysis on the highest-confidence target genes predicted by MultiCausGRN. KEGG pathway enrichment revealed significant overrepresentation of immune-related pathways, including Inflammatory Bowel Disease (FDR = 0.0013) and Th1 and Th2 Cell Differentiation (FDR = 0.0019), with additional enrichment observed for Th17 Cell Differentiation and T-Cell Receptor Signaling pathways (Figure 10). These pathways are highly relevant to PBMC biology, as peripheral blood mononuclear cells play central roles in adaptive immune responses, T-cell activation, cytokine signaling, and immune-cell differentiation (Abbas et al., 2021; Murphy and Weaver, 2022). The enrichment of immune-associated pathways suggests that the inferred regulatory interactions capture biologically meaningful transcriptional programs rather than merely reflecting statistical associations within the data.

Although the biological validation analyses support the relevance of the inferred regulatory network, these results should be interpreted within their scope. The PCA visualization of learned embeddings, hub transcription factor analysis, and KEGG/GO enrichment results primarily demonstrate that MultiCausGRN successfully recovers known immune-related regulatory programs present in PBMCs, including pathways associated with T-cell differentiation, cytokine signaling, and immune activation. These analyses provide evidence that the inferred network is biologically plausible and consistent with established immune biology; however, they do not by themselves establish the discovery of novel regulatory interactions. Independent experimental validation, such as perturbation-based studies, CRISPR screening, transcription factor knockdown experiments, or ChIP-seq–based confirmation of predicted TF–target interactions, would be required to validate newly inferred regulatory relationships. Recent perturbation-response frameworks, including CausalPert (Martell et al., 2026) and LangPert (Märtens et al., 2025), further illustrate how large-scale perturbation datasets can be used to evaluate regulatory effects following genetic interventions. While these methods are not direct baselines for MultiCausGRN, they highlight promising future directions for validating and refining inferred regulatory networks using perturbational evidence. Such experimental and perturbation-based validation was beyond the scope of the present study and remains an important direction for future work.

### 3.5 Comparison with the competitive multi-omics GRN inference methods

To position MultiCausGRN against recent multi-omics GRN approaches, we compared it conceptually and empirically with representative methods that integrate chromatin accessibility with transcriptional information, namely LINGER (Yuan and Duren, 2025), MultiGNN (Li et al., 2022), scMTNI (Zhang et al., 2023), and CellOracle (Kamimoto et al., 2023). These methods reflect different design philosophies. The comparison indicates that MultiCausGRN occupies a distinct methodological niche by combining graph attention-based regulatory learning with explicit prior integration, rather than relying primarily on external pretraining, lineage structure, or motif-constrained linear refinement. Among the compared methods, LINGER is arguably the strongest recent reference point. It infers GRNs from paired single-cell multiome data while incorporating atlas-scale external bulk data through lifelong learning and TF–RE motif knowledge through manifold regularization. The authors reported that LINGER achieved a fourfold to sevenfold relative increase in accuracy over existing methods and showed strong benchmarking performance, including binding-potential AUROC values around 0.92 and AUPRC ratio around 5.17 for representative examples. However, LINGER’s strength is tightly linked to its use of large-scale external bulk resources and a training strategy explicitly designed to retain prior information from those data during adaptation to the target multiome dataset. In contrast, MultiCausGRN does not depend on atlas-scale pretraining; instead, it improves regulatory recovery by injecting prior edges directly into the graph attention model, yielding a PBMC test AUROC of 0.682 ± 0.026 and test AUPRC of 0.743 ± 0.049 with improved stability across seeds. In addition, LINGER prioritized transfer of regulatory knowledge across broad cellular contexts, whereas MultiCausGRN prioritizes robust prior knowledge regularization within the target graph itself, which is more lightweight and potentially easier to apply when large external reference compendia are unavailable.

MultiGNN is the closest comparator in terms of modern graph-based modeling. It is a supervised GNN link-prediction framework that integrates gene expression and chromatin accessibility and was evaluated on public multi-omics datasets using BEELINE-derived ground truths. The authors reported that MultiGNN achieved the best results on nearly all tested datasets while also maintaining strong training efficiency on larger datasets. On dense datasets such as human kidney and PBMC, the authors noted particularly strong performance. Nevertheless, MultiGNN remains fundamentally a supervised feature-fusion model, and its main advantage is attributed to cross-modal fusion and link prediction under curated benchmark settings. By contrast, MultiCausGRN contributes a different form of inductive bias: rather than only fusing modalities, it imposes biologically grounded structure through prior TF–target edges from OmniPath, which improved both average AUROC/AUPRC and reduced variance across repeated runs in the PBMC experiment. Thus, whereas MultiGNN demonstrates the strength of supervised multi-omics graph learning, MultiCausGRN extends this direction toward prior knowledge regularization and interpretability, which is particularly valuable in noisy and data-limited settings.

A different comparison axis is provided by scMTNI, which was designed for cell type-specific and lineage-aware GRN inference from scRNA-seq and scATAC-seq data. scMTNI used a multi-task framework with a probabilistic lineage-tree prior, and its results showed that multi-task learning generally outperformed single-task alternatives on sparse simulated datasets. These results highlighted scMTNI’s strength in modeling dynamic regulatory transitions across cell states. However, this also clarifies a key distinction from MultiCausGRN: scMTNI is primarily a lineage-structured multi-task probabilistic framework, whereas MultiCausGRN is aimed at global graph-based regulatory recovery with prior guided constraints. Accordingly, MultiCausGRN is not trying to solve the same problem of branch-aware network evolution, but instead targets stable link prediction in multi-omics GRNs where a prioriguided edges can guide the attention mechanism toward more plausible regulatory structure.

CellOracle is another important baseline because it integrates transcriptomic and chromatin accessibility information through regulatory sequence analysis and then refines a base GRN using regularized linear models. Its main strength lies in producing interpretable, cell-state-specific GRNs and enabling *in silico* TF perturbation simulation. In validation against active regulatory elements and TF ChIP-seq data, CellOracle reported AUC values in the range of approximately 0.8–0.87 for regulatory element state prediction and around 0.7–0.8 for TF-target recovery in specific validation tasks. In the scMTNI study, CellOracle also achieved strong F-scores on ChIP and perturbation-derived gold standards, although the inferred networks contained a substantially smaller number of regulators (7–11) compared with scMTNI + Prior (29–36). This is a meaningful contrast with MultiCausGRN. CellOracle is strong in interpretability and perturbation analysis, but its reliance on a staged prior-construction-and-pruning pipeline and regularized linear modeling may limit its ability to capture more complex higher-order regulatory dependencies. MultiCausGRN instead leverages two-layer graph attention and learned embeddings, while its prior knowledge serves as a structural constraint rather than only a pre-filter, allowing it to balance predictive performance with biologically meaningful graph organization.

Taken together, these comparisons suggest that MultiCausGRN should not be framed simply as another multi-omics GNN. Relative to LINGER, it avoids dependence on atlas-scale bulk pretraining; relative to MultiGNN, it adds prior biological constraints rather than relying solely on feature fusion; relative to scMTNI, it does not require lineage structure and is better aligned with global GRN link prediction; and relative to CellOracle, it replaces predominantly linear, prior-pruned inference with attention-based non-linear graph learning. In our PBMC evaluation, this design led to a consistent improvement from 0.554 ± 0.014 to 0.682 ± 0.026 in test AUROC and from 0.590 ± 0.163 to 0.743 ± 0.049 in test AUPRC after incorporating prior knowledge, while also reducing run-to-run variability. Therefore, the main contribution of MultiCausGRN is not merely competitive predictive accuracy, but the demonstration that prior edges integration can improve robustness, stability, and biological plausibility in graph-based multi-omics GRN inference. Overall, MultiCausGRN complements existing multi-omics GRN methods by showing that prior knowledge can be incorporated directly into graph attention learning to improve robustness and interpretability, without requiring atlas-scale external pretraining or lineage-specific modeling. Table 5 explains the main core values and limitations, and differences between MultiCausGRN and the competitive models.

**TABLE 5:** Comparison of representative multi-omics GRN inference methods and the proposed MultiCausGRN model.

Method	Omics used	Core strategy	Use of prior knowledge	Strengths	Limitations/ Difference from MultiCausGRN
LINGER	scRNA + scATAC + external bulk atlas	Lifelong neural network with manifold regularization and transfer learning	TF motif prior and atlas-scale bulk data	High accuracy improvement, enables TF activity inference and GWAS interpretation	Requires large external reference datasets and complex pretraining; less focused on prior knowledge graph regularization
MultiGNN	scRNA + scATAC	Supervised graph neural network link prediction framework	Implicit via curated training labels and feature fusion	Strong predictive performance across multiple datasets; captures multi-omics dependencies	Requires labeled regulatory edges and benchmark-specific preprocessing; focuses on feature fusion rather than prior knowledge structure
scMTNI	scRNA + scATAC	Multi-task probabilistic graphical model with lineage-tree prior	Cell-type-specific motif-derived prior networks	Effective modeling of dynamic GRN evolution and cell lineage transitions	Designed for lineage-aware inference rather than global network recovery; scalability to large gene sets may be limited
CellOracle	scRNA + scATAC + regulatory sequence data	Two-stage inference: motif-based base GRN followed by regularized linear refinement	Strong reliance on motif-derived regulatory sequence prior	Interpretable GRNs and enables *in silico* TF perturbation simulation; reported ROC AUC ∼0.7–0.87	Linear modeling limits capture of complex nonlinear interactions; staged pruning may restrict network coverage
MultiCausGRN	scRNA + scATAC + prior regulatory network (TRRUST)	Graph attention network with prior edge integration	Explicit TF–target prior incorporated during training	Improved robustness and stability (PBMC AUROC 0.682 ± 0.026, AUPRC 0.743 ± 0.049; balances predictive performance and biological interpretability	Depends on quality of prior network; does not explicitly model lineage dynamics or use large external atlases

To further evaluate the effectiveness of MultiCausGRN, it was compared with the competitive representative GRN inference approaches: MultiGNN, CellOracle, and scMTNI, using the same PBMC multi-omics dataset and identical OmniPath-derived train, validation, and test splits. To improve the transparency of the comparative evaluation, we standardized all baseline inputs using the same processed PBMC multi-omics dataset and the same training parameters. This ensured a fair comparison across all methods under the same evaluation setting. Due to substantial computational and environment dependencies associated with atlas-scale external pretraining, LINGER was retained as a qualitative comparison only because its dependency and reference-data requirements prevented a stable end-to-end PBMC run under the same evaluation protocol. For MultiGNN, following its preprocessing approach, the RNA matrix was paired with the ATAC gene-activity matrix after gene-level alignment; duplicate ATAC genes were averaged, and genes without ATAC signal were assigned zero-valued accessibility features. TF indices were mapped to gene names using the same TF list used in MultiCausGRN, and the model was run for 90 epochs with additive reduction. For scMTNI, the work was at a higher level; PBMC cells were grouped according to cluster labels, cluster-specific expression tables were generated, and regulator files were constructed from the same TF list. scMTNI was then executed using the prepared PBMC configuration files and the parameter settings reported in Table 6. CellOracle was evaluated as a prior-guided baseline because its workflow constructs and refines GRNs using regulatory prior information; the same TRRUST prior file used to generate the MultiCausGRN is used to maintain the results. To maintain computational feasibility, all competing baseline methods were evaluated using the same PBMC train/validation/test split and a representative random seed (seed = 2), whereas MultiCausGRN was additionally evaluated across five independent random seeds (1, 2, 4, 8, and 16) to assess robustness and performance variability. Therefore, the baseline comparisons in Table 6 correspond to single-seed evaluations, while the multi-seed analysis is reported separately in Table 3. Method-specific input files were generated from the common PBMC expression matrix, TF list, and chromatin accessibility data to ensure a consistent evaluation protocol while preserving the original requirements of each implementation. The proposed MultiCausGRN framework consistently achieved the best overall performance in terms of AUPRC and a competitive performance in terms of AUROC. The incorporation of prior knowledge substantially improved the predictive capability of the model compared to the baseline configuration, demonstrating the importance of integrating biologically grounded regulatory information into graph-based GRN inference. MultiGNN achieved competitive performance; however, its results remained below those of MultiCausGRN, likely because MultiGNN primarily focuses on integrating multi-omics features without explicitly modeling prior knowledge regulatory dependencies. Similarly, CellOracle and scMTNI demonstrated reasonable predictive capabilities but showed reduced performance compared to the proposed framework, potentially due to limitations in modeling global graph dependencies and prior knowledge interactions simultaneously. Overall, these results indicate that combining GAT mechanisms with prior-guided learning enables MultiCausGRN to learn more biologically meaningful regulatory representations and improves the reliability of inferred transcription factor–target interactions in complex scRNA and scATAC data.

**TABLE 6:** Comparative evaluation of MultiCausGRN and competing GRN inference frameworks on the PBMC multi-omics dataset using the same OmniPath-derived train/validation/test split.

Method	Omics used	PBMC preprocessing	Prior-guided knowledge	Hyperparameters	AUROC	AUPRC
MultiGNN	scRNA + scATAC	MultiCausGRN ExpressionData were used as RNA input. ATAC gene-activity matrix was loaded from PBMC ATAC gene activity list, transposed to genes × cells, duplicate gene names were averaged, and ATAC was aligned to the RNA gene universe. Missing ATAC genes were filled with zero values. MultiCausGRN TF indices from were converted to TF names	NO	epochs = 90, seed = 2; reduction = add, seed = 2	0.65	0.62
scMTNI	scRNA + scATAC	PBMC cells were grouped by cluster labels. For each cluster, a gene × cell expression table was generated from the MultiCausGRN Expression file. TF names were derived from the same TF list used by MultiCausGRN. scMTNI input files were generated using PreparescMTNIinputfiles.py in its GitHub repository	TRRUST regulatory prior	Epochs = 90, seed = 2, splitgene = 50, motifs = 0, x = 50, p = 0.2, c = yes, b = −0.9, q = 0	0.69	0.73
CellOracle	scRNA + scATAC	MultiCausGRN expression matrix was converted to AnnData format, normalized (target_sum = 10,000), and log-transformed. The MultiCausGRN TRRUST prior network was used as the base GRN. Only TF-target pairs present in the PBMC gene set were retained	TRRUST regulatory prior + CellOracle regression refinement	PCA, n_pca_dims = 30, KNN imputation (k = 30, balanced = True, b_sight = 30, b_maxl = 200), GRN fitting (alpha = 10), edge filtering (p < 0.05, weight = coef_abs), epochs = 90, seed = 2	0.67	**0.70**
MultiCausGRN (Baseline)	scRNA + scATAC	Same PBMC RNA and ATAC matrices after quality control, feature selection, and TF-target mapping. Multi-omics features were integrated through graph-based representation learning	No	epochs = 90, seed = 2	**0.703**	0.686
MultiCausGRN	scRNA + scATAC	Same PBMC RNA and ATAC matrices after quality control, feature selection, and TF-target mapping. Multi-omics features were integrated through graph-based representation learning	TRRUST regulatory prior	epochs = 90, seed = 2	**0.712**	**0.782**

MultiCausGRN consistently achieved the highest AUROC and AUPRC values, demonstrating the effectiveness of integrating prior knowledge with multi-omics graph learning. Bold values indicate the best performance among all compared methods.

## 4 Conclusion

In this study, we proposed MultiCausGRN, a prior-guided graph attention framework for gene regulatory network inference from multi-omics data. Unlike conventional GRN inference approaches that rely primarily on expression correlations, motif-based pruning, or large-scale external pretraining, the proposed model integrates biologically grounded regulatory priors directly within the graph learning process. This design enables the model to capture nonlinear regulatory dependencies while preserving interpretable prior-guided structure in the inferred networks.

Experimental results on the PBMC multi-omics dataset demonstrate that incorporating prior information improves both predictive performance and robustness. Specifically, MultiCausGRN achieved a mean test AUROC of 0.682 and a mean test AUPRC of 0.743, outperforming the similar approaches and reducing variability across repeated runs. These findings suggest that prior-guided graph attention learning can enhance stability in realistic GRN inference settings characterized by noise, sparsity, and limited sample size. Comparison with recent multi-omics GRN inference methods further highlights the complementary contribution of the proposed model. The proposed method benefits from attention-based nonlinear modeling while maintaining biological interpretability through prior edge integration.

Despite these advantages, several limitations remain. The performance of MultiCausGRN depends on the quality and coverage of the prior regulatory network, and the MultiCausGRN framework does not explicitly model dynamic regulatory changes across cell lineages. Future work will explore adaptive prior weighting, integration of additional omics modalities such as proteomics or epigenetic marks, and extension toward temporal and disease-specific GRN inference. Furthermore, large-scale benchmarking across diverse datasets will be essential to fully characterize the generalizability of prior-guided graph learning strategies in regulatory network reconstruction. In addition, although MultiCausGRN incorporates biologically curated directional regulatory priors to guide graph learning, the current framework does not perform formal causal inference or causal effect estimation. Future work will investigate the integration of perturbation-based datasets, CRISPR screening data, and intervention-aware learning strategies to enable more rigorous assessment of causal regulatory mechanisms. Such extensions may facilitate the validation of predicted regulatory interactions under controlled perturbations, provide stronger evidence for causal regulatory relationships, and evaluate the biological validity of inferred regulatory networks. These analyses may provide complementary evidence for the functional significance of the predicted transcription factor–target relationships. Future work will also investigate the application of MultiCausGRN to disease-focused settings, including the interpretation of disease-associated variants, disease-gene prioritization, and the identification of regulatory mechanisms underlying rare genetic disorders. The integration of inferred regulatory networks with genomic and clinical evidence may provide additional insights into disease-associated regulatory processes.

Overall, this study demonstrates that incorporating prior biological knowledge into graph neural network architectures represents a promising direction for improving both the accuracy and interpretability of multi-omics GRN inference. The proposed MultiCausGRN model contributes toward more reliable regulatory hypothesis generation and may support downstream applications in systems biology, precision medicine, and rare disease research.

## Data Availability

The datasets analyzed in this study are publicly available. The PBMC multi-omics dataset can be accessed from the 10x Genomics data repository. Benchmark gene regulatory network datasets were obtained from the BEELINE evaluation framework. Curated transcription factor–target regulatory interactions were retrieved from the OmniPath database. The data presented in the study are deposited in the Zenodo repository, accession number 10.5281/zenodo.20377752. The source code and processed datasets supporting the conclusions of this study are publicly available at the following GitHub link: https://github.com/nrr-90/MultiCausGRN.
