# Supplementary material for: MultiCausGRN: directed prior-guided graph attention model for multi-omics gene regulatory network inference
Source: Front Bioinform. 2026 Aug 4;6:1883130. doi: 10.3389/fbinf.2026.1883130 (PMC13481796; doi:10.3389/fbinf.2026.1883130)
Supplement: Supplementary file 1 [file DataSheet1.pdf]

Supplementary Material
MultiCausGRN: Directed Prior-Guided Graph Attention Model for Multi-Omics Gene Regulatory Network Inference

Noor Jamal Alkhateeb
Mamoun Awad

1 Supplementary Material

This supplementary material provides additional implementation details, dataset preprocessing information, and robustness analyses supporting the results presented in the main manuscript.

Table 1: Implementation details and hyperparameters used for all MultiCausGRN experiments.

**Parameter**	**Value**
Framework	PyTorch
Optimizer	Adam
Learning rate	0.003
Epochs	90
Batch size	256
Learning-rate scheduler	StepLR
Scheduler step size	1 epoch
Scheduler decay factor (γ)	0.99
Attention heads (Layer 1)	3
Attention heads (Layer 2)	3
Hidden dimensions	[128, 64, 32]
Output embedding dimension	16
Attention activation	LeakyReLU
LeakyReLU coefficient (α)	0.2
Dropout rate	0.01
Aggregation strategy	Concatenation
Decoder	Dot product
Graph type	Directed graph
Self-loops	Disabled
Input normalization	StandardScaler (z-score)
Weight initialization	Xavier Uniform
Model selection criterion	Best validation AUPRC
Random seeds	1, 2, 4, 8, 16
Hardware	NVIDIA GPU (CUDA)

Table 2: PBMC dataset preprocessing and benchmark construction.

**Step**	**Description**
Input dataset	PBMC multiome (scRNA-seq + scATAC-seq)
Gene filtering	Low-expression genes removed
HVG selection	Top 5000 highly variable genes
Retained genes	3718 genes
Transcription factors	153 candidate TFs
Benchmark TFs	24 TFs after OmniPath filtering
Benchmark targets	168 target genes
Positive interactions	224 OmniPath regulatory edges
Negative sampling	Random TF-target pairs absent from positives
Split ratio	70% train, 15% validation, 15% test
Identifier mapping	Harmonized gene symbols across resources
Adjacency construction	Positive training/prior edges only

Table 3: Bootstrap confidence intervals for the primary balanced PBMC test set (seed 2).

Metric	Score	Lower 95% CI	Upper 95% CI
AUROC	0.712	0.574	0.837
AUPRC	0.782	0.643	0.887

Table 4: Performance of MultiCausGRN across five independent random seeds on the balanced PBMC test set.

Seed	AUROC	AUPRC
1	0.658	0.658
2	0.712	0.782
4	0.656	0.74
8	0.680	0.761
16	0.703	0.773
Mean ± SD	0.682 ± 0.026	0.743 ± 0.049
